# Reflex Neuralgia by Foreign Body in the Ear

**Published:** 1881-10

**Authors:** 


### REFLEX NEURALGIA CAUSED BY FOREIGN BODY IN THE EAR.

The following report of a case by Dr. C. F. Arney, of Altoona, Pa., we take from the *Medical Summary*, for July, 1881:—

Sadie C. came to me July 25th, complaining of a very severe pain in her left ear and temple, paroxysmal in character, shooting from the ear to temple, and sometimes as far as the forehead. The pain usually came on as soon as she arose in the morning, and continued until she would lie down, when it would at once cease. She also complained of a sensation of fullness and impairment of hearing in her left ear. She has had more or less pain in her ear ever since she can remember. Her last attack commenced about three months ago, and has continued about the same ever since.

With the aid of a speculum, I saw the ear was filled with hardened cerumen, which I at once removed, by means of a syringe and warm water. After the wax was removed, I noticed a button sticking transversely in the external auditory meatus, so tightly that I was unable to remove it with a syringe, so I passed the hooked end of Prof. Gross's ear scoop behind it and drew it out, to the great surprise of my patient,, who said, "I do not see how that ever got in there." I concluded, from the history of the case and size of button, that it had been in the ear for a long time.

